# Progranulin and GPNMB: interactions in endo-lysosome function and inflammation in neurodegenerative disease

**DOI:** 10.1186/s12974-023-02965-w

**Published:** 2023-11-30

**Authors:** Drew A. Gillett, Rebecca L. Wallings, Oihane Uriarte Huarte, Malú Gámez Tansey

**Affiliations:** 1 Center for Translational Research in Neurodegenerative Disease (CTRND), University of Florida, Gainesville, FL USA; 2 Department of Neuroscience, University of Florida, Gainesville, FL USA; 3 McKnight Brain Institute, University of Florida, Gainesville, FL USA; 4 Norman Fixel Institute for Neurological Diseases, University of Florida, Gainesville, FL USA

**Keywords:** Progranulin, GPNMB, Endo-lysosomal system, Immune system, Neurodegenerative diseases

## Abstract

**Background:**

Alterations in progranulin (PGRN) expression are associated with multiple neurodegenerative diseases (NDs), including frontotemporal dementia (FTD), Alzheimer’s disease (AD), Parkinson’s disease (PD), and lysosomal storage disorders (LSDs). Recently, the loss of PGRN was shown to result in endo-lysosomal system dysfunction and an age-dependent increase in the expression of another protein associated with NDs, glycoprotein non-metastatic B (GPNMB).

**Main body:**

It is unclear what role GPNMB plays in the context of PGRN insufficiency and how they interact and contribute to the development or progression of NDs. This review focuses on the interplay between these two critical proteins within the context of endo-lysosomal health, immune function, and inflammation in their contribution to NDs.

**Short conclusion:**

PGRN and GPNMB are interrelated proteins that regulate disease-relevant processes and may have value as therapeutic targets to delay disease progression or extend therapeutic windows.

## Background

A significant challenge in the field of human health is neurodegenerative disease (ND). An estimated 4.7–6 million individuals in the United States alone are diagnosed with some form of a ND, presenting a significant burden on both caregivers and patients alike [1–5]. Medication to address symptoms of NDs exists, but disease-modifying therapeutics to reverse or delay disease progression do not yet exist. This makes the identification and manipulation of therapeutic targets a critical pre-requisite for advancing the development of effective treatment strategies. The specific symptoms and progression of NDs can vary widely, but a unifying and newly recognized feature of the most prevalent NDs, including Parkinson’s disease (PD), Alzheimer’s disease (AD), and Frontotemporal dementia (FTD), is the role of the immune system and central–peripheral neuroimmune crosstalk in both the etiology and progression of ND. Two proteins in particular have been highlighted by analysis of ND-associated genes: progranulin (PGRN) and glycoprotein non-metastatic B (GPNMB) [6–15]. Furthermore, recent evidence supports that a loss of PGRN results in an increase in GPNMB expression [17, 18]. The mechanistic relationship between these two proteins, as well as the potential phenotype associated with increased GPNMB, has not been elucidated. The purpose of this review is to discuss the field’s current understanding of these two critical and interrelated proteins, with a focus on their roles in endo-lysosomal health and immune cell function.

### Progranulin and GPNMB biology

#### Progranulin structural biology

**Fig. 1:**
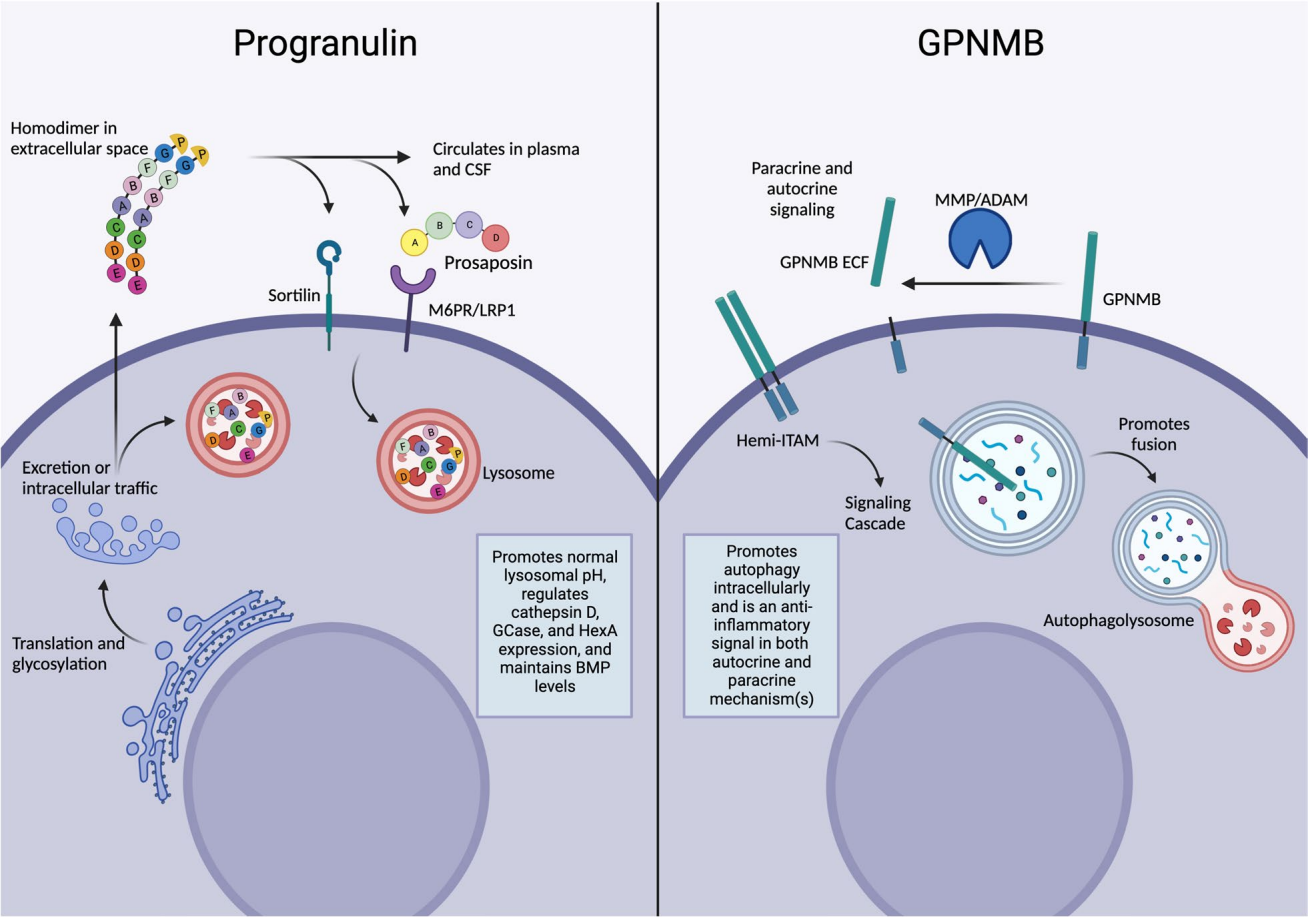
Diagram of the cell biology of progranulin and GPNMB. Figure created with BioRender.com

The structure of PGRN has been described as “beads on a string”, where individual granulins (GRNs), the cysteine-enriched repeats that compose full-length PGRN, are connected together with short linker regions [19]. Once translation has been completed, the nascent PGRN is folded in the endoplasmic reticulum (ER), where the cysteine residues form disulfide bonds promoting the beta-folds in the GRNs and glycosylation of PGRN in both the ER and the Golgi at five asparagine loci: 118, 236, 265, 368, and 530. From the Golgi body, PGRN can be trafficked directly into the endo-lysosomal pathway or secreted out of the cell and allowed to circulate in biofluids (Fig. 1).

To be internalized, PGRN can bind to one of its receptors, sortilin, using a C-terminus motif near granulin 7 (granulin E) [20], or the C/D GRNs can bind to the BC linker region of prosaposin and piggy-back into the cell via prosaposin receptors, M6PR and LRP1 [21]. Once internalized, PGRN localizes to the endo-lysosomal system, where it is processed into individual, 6–8 kDa GRNs by lysosomal proteases, including multiple cathepsin species [22, 23], where they remain stable for a considerable but undetermined length of time [24]. In the extracellular space, PGRN can also be cleaved into GRNs by several matrix metalloproteases, such as MMP12 [25], ADAMST 7 and 12 [26], proteinase 3 [27], and elastase [27]. In contrast, secretory lymphocyte precursor inhibitor (SLPI) protects PGRN from proteolytic cleavage and allows it to persist in the extracellular space [28]. However, inhibition of PGRN uptake also decreases the extracellular GRNs concentration, strongly suggesting that GRNs are primarily generated by PGRN degradation in the lysosome [29].

Mature, secreted PGRN is a homodimer in the extracellular space, and it is this form that circulates in the plasma of both humans and mice [30]. Healthy individuals have an average of 96–125 ng/mL of blood PGRN [31, 32]. Altered levels of plasma PGRN can be used both as a diagnostic biomarker and a predictive variable for disease severity and/or progression [33, 34].

#### Progranulin expression

PGRN is expressed at varying levels in multiple cell types throughout the body, including neurons, muscle cells, endothelial cells, and adipocytes. Interestingly, myeloid cells, including microglia, macrophages, and monocyte-derived dendritic cells, express higher levels PGRN than most other cells [35, 36]. The amount of PGRN expression varies among myeloid cells residing in different body compartments, suggesting the cellular micro-environment modulates the expression, and several studies indicate that myeloid cells increase PGRN expression in response to immunological challenges [37, 38].

PGRN is necessary for healthy aging, but there are limited data to suggest that PGRN expression changes over the course of an organism’s lifespan, and genetic factors are far more likely to influence PGRN levels. Various frameshift, nonsense, and deletion mutations in the *GRN* gene exist in the human population, but the most common FTD-associated mutation in *GRN* is *R493X*, where an arginine codon is replaced with a termination signal [39]. This results in less PGRN being produced from the affected allele, as both the mRNA and protein product are degraded by nonsense-mediated decay (NMD). A single defunct allele results in less than 50 ng/mL of PGRN in blood, which is less than half of the average circulating level of PGRN in healthy controls, 96–125 ng/mL [31, 32].

Several other FTD-associated mutations result in the same effect, but three other major mechanisms of PGRN deficiency exist: a mutation in the signal recognition peptide (SRP), 5’ untranslated region (UTR) mediated suppression, and DNA methylation. The swap of an alanine to an aspartic acid at position 9 (A9D) in the nascent protein interferes with the signal recognition peptide (SRP) that successfully traffics PGRN to the ER. The resulting PGRN protein lacks the appropriate environment for protein folding and post-translational modifications (PTM) and is not viable [40]. Another method involves the 5’ UTR-mediated regulation of *GRN* mRNA. A previous study identified a novel mutation in the splice site of the 5’ UTR region of PGRN in FTD patients from the same family [41]. No other FTD-associated genes were abnormal, and a test for NMD was negative, yet the splice site mutation resulted in roughly half as much PGRN [41]. Subsequent work identified that the upstream open reading frame (uORF) elements within the 5’ UTR interfere with both the efficiency and the stability of the *GRN* mRNA, resulting in less PGRN protein being produced [42], but it remains unclear what factors determine the inclusion or exclusion of the longer 5’UTR. Finally, the degree of DNA methylation can epigenetically determine the expression of the *GRN* gene. Higher amounts of *GRN* DNA methylation result in less PGRN mRNA and protein [43].

One final mechanism regulates PGRN expression but does not rely on changes to the *GRN* gene itself. Micro RNAs (miR) are small pieces of RNA that modulate mRNA expression. The *GRN* gene has three miRs that bind to the 3’ UTR region and repress its expression [44–46]. Understanding the factors that regulate PGRN expression is important given the known association between alterations in PGRN expression and NDs and the potential of targeting these factors to modulate PGRN expression.

#### GPNMB structural biology

GPNMB is a glycosylated type-1 transmembrane protein that is localized to both the cell membrane and to components of the autophagy pathway. Originally identified in a low-metastatic cell line in 1995 [47], GPNMB was later found to not necessarily correlate with low metastatic activity and instead has been investigated for its ability and shown to promote metastasis in some mice models [48–50]. GPNMB is a member of the PMEL/NMB family and has a large extracellular domain (ECD) that includes a cell-attachment site at a.a. 64–66 (RGD), a PKD domain at 240–327, and a disordered region at 320–362 [51]. GPNMB is expressed as two isoforms generated from alternative splicing, which determines whether 12 amino acids are included or excluded at aa 339–340 [52, 53]. No distinct function has been ascribed to either isoform, but the modification of a disordered region may allow or preclude different protein–protein interactions. The short intracellular domain is only 53 amino acids, and it contains a half immunoreceptor tyrosine-activation motif (hemITAM) and a dileucine motif [51]. GPNMB is heavily glycosylated, with 12 asparagine sites for N-linked glycosylation [51]. Nascent GPNMB has a molecular weight of approximately 65 kDa, but the glycosylated forms have a weight of around 90 kDa and 115kDa [53]. The 115 k Da species of GPNMB is localized to the cell membrane and is susceptible to ECD cleavage by ADAM10 [54], but the 90 kDa species is localized to the endo-lysosomal pathway. All glycosylated GPNMB is subject to serine phosphorylation (S542), but only the fully mature 115 kDa species can be cleaved to generate the ~ 100 kDa GPNMB extracellular fragment (ECF) (sometimes called soluble GPNMB (sGPNMB)) [53, 54] (Fig. 1).

#### GPNMB expression

While low levels of expression exist in numerous cell types, GPNMB is primarily expressed by osteoclasts, osteoblasts, melanocytes, endothelial cells, myeloid cells, and antigen-presenting cells (APCs), such as macrophages and dendritic cells [48], which means GPNMB is expressed in multiple compartments throughout the body. The GPNMB ECF is also present in biofluids, including circulating in blood serum [55, 56] and urine [57]. Myeloid cells and astrocytes in the central nervous system (CNS) can secrete GPNMB ECF into the cerebrospinal fluid (CSF) [58–60]. At rest, control participants had an average blood serum GPNMB ECF of 31 ± 4.9 ng/mL [56], but the cleavage of GPNMB ECF is dynamic and can reflect the health of a patient, including immune cell activity [61–63], general organ health [64], and altered metabolism and/or endocrine function [56]. The regulation of GPNMB expression in immune cells has not been fully characterized, but inhibition of microphthalmia-associated transcription factor (MITF) activity resulted in decreased GPNMB expression in RAW264.7 cells [65], dendritic cells [66], and osteoclasts [67]. The *MITF* gene can be alternatively spliced into multiple isoforms of MITF [68], but it is unclear if any isoform(s) control GPNMB expression to a greater degree than the others.

GPNMB was also identified as a gene the expression of which is increased with aging in bone-marrow mesenchymal stem cells (bmMSC) collected from human subjects [69] and skeletal muscle macrophages in mice [70]. However, it is unclear if this increase is beneficial or deleterious. Vaccination against GPNMB significantly increased lifespan in a progeroid (*Zmpste24* KO) mouse model of aging [71]. This approach could not distinguish between cell types, but the removal of GPNMB-positive cells was beneficial in this model, suggesting that GPNMB has a role in aging that has not been fully elucidated.

### PGRN and GPNMB biological functions

#### PGRN

As described previously, PGRN localizes to organelles in the endo-lysosomal system and is processed into individual GRNs by lysosomal proteases. Within the lysosome, PGRN deficiency has been linked to an increase in lysosomal pH [72, 73], but there is no change in the amount of vacuolar-type-H + ATPase (V-ATPase) present in cells with and without sufficient PGRN [73]. Regardless of the precise mechanism, the increase in lysosomal pH and, therefore, decrease in available H + ions would decrease the ability of acidic lysosomal enzymes to function, which in turn increases the expression of those lysosomal enzymes [72, 73]. Indeed, insufficient PGRN dysregulates cathepsin D expression [74–77], beta-glucocerebrosidase (GCase) [78–80], and B-hexosaminidase A (HEXA) [81]. Each of these enzymes has an important role in lysosomal metabolism, and like PGRN, a loss of function in these enzymes is associated with lysosomal storage disorders (LSDs).

Another underrepresented facet of lysosome biology that is related to PGRN is lipid metabolism. Multiple groups have identified altered lipid metabolism in the cortex of mouse progranulin (mPGRN)-deficient mice [82, 83] and in FTD patient plasma [84]. The precise mechanism is not fully understood, but a lack of mPGRN has also been associated with decreased amounts of bis(monoacylglycerol)phosphate (BMP) [82]. BMP is a phospholipid that exists exclusively in the lysosome, where the negatively charged phosphate group is believed to act as a raft or a dock for lipid degrading enzymes such as saposins B and C, acid sphingomyelinase (ASM), lysosomal phospholipase A2 (LPLA2), and GCase [85–88] that promotes their activity and allows lysosomal lipid metabolism to occur. BMP also interacts with apoptosis-linked gene 2 interaction protein X (ALIX), Niemann–Pick disease type C2 protein (NPC2), and heat shock protein 70 (HSP70) [89–91]. These BMP-binding partners also play a role in endo-lysosomal membrane and sorting dynamics by altering the curvature and cholesterol trafficking of the endo-lysosome membrane [92, 93]. Further work is needed to determine the extent to which PGRN-deficient lipid metabolism is mediated by BMP and/or through other pathways.

Within the brain, PGRN is largely associated with neurogenesis and neuroprotection. Five days after sustaining a closed-cortical impact, *Grn* knock-out (KO) mice developed increased perilesional axonal injury relative to wild-type (WT) controls despite having the same size lesion and a similar number of IBA + myeloid cells in the area [38]. qPCR from RNA collected from the ipsilateral side of the injury revealed increased mRNA of inflammatory markers, including *Il-1β*, *Il-6*, and *Tnf,* in the *Grn* KO mice relative to WT controls but decreased mRNA for the anti-inflammatory *Il-10* [38]. In a stroke model, WT mice underwent surgery to permanently block their middle cerebral artery (pMCAO) and, 30 min later, were administered recombinant PGRN directly into the right ventricle (ICV). Mice that received a single dose of PGRN showed increased numbers of proliferating neuronal stem cells (NSCs) in the subgranular zone of the dentate gyrus relative to both mice that did not receive PGRN or had a sham surgery [94]. The same PGRN treatment paradigm also rescued anxiety-like phenotypes and cognitive deficits that were apparent in the pMCAO mice [94]. Finally, while there is no genetic association between *GRN* and ALS, PGRN immunopositivity is increased in microglia found in the spinal cord of ALS patients relative to controls [95, 96]. A similar increase in *Grn* expression is noted during the progression of symptoms in mouse models of ALS [97].

Collectively, it seems that PGRN expression is beneficial in the CNS, with a loss of PGRN being deleterious in multiple models of CNS injury or neurodegeneration. Outside of the brain, the loss of PGRN impacts various organs and peripheral compartments differently, but the peripheral immune system has been reported to be significantly impacted by a decrease in PGRN expression. Human *GRN*-mutation carriers with clinically symptomatic FTD symptoms were reported to have increased plasma levels of sCD163 and CCL18, both of which are peripheral myeloid cell markers, suggesting an increase in peripheral immune activity relative to asymptomatic *GRN*-mutation carriers and healthy controls [98]. This also highlights the importance of compensatory mechanisms during aging in *GRN*-mutation carriers, as asymptomatic *GRN*-mutation carriers that were, on average, 7.6 years younger than the symptomatic *GRN*-mutation carriers, had immune profiles that were not significantly different from healthy controls [98]. Furthermore, another marker of peripheral immune activity and inflammation, lipopolysaccharide-binding protein (LBP), was increased in *GRN*-mutation carriers and correlated with white-matter changes in the frontal cortex of both symptomatic and asymptomatic *GRN*-mutation carriers [98], suggesting there is a spectrum of peripheral immune activation and CNS changes that exist prior to clinical diagnoses.

Macrophages from mouse models of PGRN deficiency also exhibit altered responses to lipopolysaccharide (LPS), including increased transcription of *Mcp-1*, *Il-12p40*, and *Tnf,* as well as decreased transcription of *Il-10* relative to WT controls [99]. This is consistent with a greater pro-inflammatory response in *Grn* KO macrophages than in WT controls. *Grn* KO mice also demonstrated increased vulnerability to challenge by *Listeria monocytogenes* despite their increased pro-inflammatory activity [99]. In-depth investigation into the phenotypes of peripheral and central immune cell populations of 20-month-old *Grn* KO mice revealed sex-specific differences, including a higher frequency of Ly6C high monocytes in the blood of *Grn* KO females relative to WT females [100]. Specifically, *Grn* KO males showed a similar increase in MHCII, but it did not reach statistical significance. In the brain, *Grn* KO females displayed decreased MHCII expression on microglia compared to WT controls, while males showed no difference regardless of genotype. There was also a difference in T-cell populations, with *Grn* KO males exhibiting an increase in peripheral blood CD8 + T cells relative to WT controls, with no significant difference observed in *Grn* KO females [100]. In addition, CD44 expression was altered in T-cell populations: CD4 + T cells expressed less CD44 in the blood of *Grn* KO females, and the loss of PGRN abrogated the sex-dependent difference of CD44 expression on CD8 + T cells in the blood of WT controls [100]. In the brain, *Grn* KO males had increased counts of CD8 + T cells relative to WT controls, while *Grn* KO females had no change in CD8 + T cells relative to controls [100]. It is unclear what mechanism(s) are behind the sex differences of these immune cell populations, although modulation of *Grn* expression in the rodent brain by sex hormones has been reported [101], and in humans, there are known sex differences in the prevalence of neurodegenerative conditions, such as FTD–*GRN*, AD, and PD [102–104]. Altogether, it is reasonable to expect that mutations impacting PGRN levels may have differential sex-dependent immunomodulatory effects.

Although full-length PGRN in the extracellular matrix was reported by one group to interact directly with TNF receptors 1 and 2 (TNFR1/2) in a fashion that competed with native TNF binding for its own receptors [105], several other groups were unable to replicate this effect using multiple assays and experimental approaches [106–108]. Recently, new evidence has suggested that another protein, Y-box-binding protein (YB-1), may interact with both progranulin and TNFR, potentially explaining the discordant results [109]. YB-1 has several notable intracellular effects, but the protein can also exist outside of the cell, where its function is less understood [110, 111]. As an example, the addition of a combination of YB-1 and full-length mPGRN blunted the TNF-mediated inflammatory effect on bone-marrow-derived macrophages (BMDMs) by showing decreased phosphorylated nuclear factor kappa-light-chain enhancer of activated B cells (pNFkB), which is a critical transcription factor for inflammatory responses [109]. Further work on mPGRN–YB-1 interactions with TNFR will be needed to determine the kinetics of inflammatory modulation and the extent to which YB-1-enhancing therapy could be a viable approach to PGRN insufficiency.

##### GPNMB

The precise role of GPNMB in the phagolysosome is unclear, but evidence generated from epithelial cells and macrophages suggests that GPNMB aids in LC3 recruitment to the phagosome, thereby allowing phagosome fusion to the lysosome [112]. However, the majority of LC3 + /GPNMB + vesicles observed were not double-membrane vesicles, which is the signature of autophagosomes [112]. GPNMB also appears to have a role in signaling and contains a conserved hemi-ITAM domain (YxxI) on the C-terminus [51]. Src, a tyrosine kinase, phosphorylates this domain to activate downstream effects [113]. The precise effects are likely cell-dependent, but in an epithelial cell line, the expression of the GPNMB ITAM mutant (GPNMB YF) leads to a significant decrease in cell migration and sphere formation, which is consistent with the epithelial–mesenchymal transition (EMT), a process related to embryogenesis, wound healing, and cancer metastasis [113–115]. It remains unclear the extent to which the hemi-ITAM on GPNMB contributes to downstream effects in other cell types.

The role of GPNMB in the CNS is less understood, but it has been implicated in development. An early mouse model harboring a mutant *Gpnmb* allele develops pigmentary glaucoma between 1 and 3 months of age, but the presence of a functional *Gpnmb* allele prevented the development of glaucoma entirely [116]. IBA1 staining of the retina and optic nerve of D2 mice revealed increased numbers of IBA1 + cells at 1, 3, and 5 months of age relative to D2G mice [116]. This supports the argument that mutant GPNMB, specifically in IBA1 + myeloid cells, promotes the mechanism(s) of pigmentary glaucoma to occur at a young age, but a single functional allele of *Gpnmb* prevents the development of glaucoma.

Interestingly, GPNMB expression has also been reported to increase in CNS disorders. *Hexb*^*−/−*^ mice, a model of Sandhoff disease, have a significantly increased amount of *Gpnmb* transcript relative to *Hexb*^+/−^ mice [117]. In addition, immunohistochemical staining shows an increase in GPNMB-positive signal in both the thalamus and the brainstem relative to *Hexb*^+/−^ mice [117]. However, both the increase in transcript and immunohistochemical staining can be rescued with the inhibition of glucosylceramide synthase (GCS), the enzyme that produces beta-D-glucosylceramide, the precursor to the sphingolipid species that accumulate in Sandhoff disease [117]. GPNMB expression also increases with neuroinflammation. Relative to non-neurological disease (NND) controls, Neuro-HIV brains showed an increase in GPNMB + IBA1 + cells in the brain [118]. This increase in *GPNMB* expression also correlated with an increase in disease-associated associated microglia (DAM) signature proteins, including *HLA-dr*, *LGALS3*, and *CTSB*, which is believed to influence the rate of synaptic pruning in Neuro-HIV patients [118]. The role(s) of GPNMB is still under investigation, but it is clear that GPNMB is regulated in response to changes in CNS health.

GPNMB has a well-documented effect on T cells, in both membrane-bound and cleaved forms, via interaction with the syndecan-4 receptor [119]. Syndecan-4 is expressed on both endothelial cells and T cells, but GPNMB exerts a cell-specific on them. Upon binding syndecan-4, membrane-bound GPNMB modulates the ability of naïve T cells to enter the S phase and produce Il-2, effectively inhibiting the primary response; and GPNMB also decreases re-activation of previously activated T cells [120]. Specifically, in oxazolone-sensitized mice, the supplementation of intravenous GPNMB ECD selectively inhibited the infiltration of T cells [121]. Instead of successfully infiltrating the inflamed skin, T cells were bound by endothelial cells that also expressed syndecan-4 [121], suggesting that the GPNMB–Syndecan-4 axis is responsible for physically orchestrating this critical immune response.

GPNMB ECD binds to CD44 and prevents the phosphorylation and translocation of NFkB in macrophages, which decreases the expression of inflammatory genes, such as *Tnf*, *Il-1α*, *Il-1β*, and *Ccl2* [122]. A similar effect has been reported to occur in microglia and astrocytes [123]. Blocking the CD44 receptor prevented the effect of GPNMB ECD on modulating inflammation [122]. GPNMB ECD has also been observed to bind the alpha-1 and alpha-3 subunits of the Na + /K + ATP-ase (NKA) in NSC-34 cells, a motor-neuron-like cell type [124]. NSC-34 cells treated with GPNMB ECF showed an increase in cell membrane potential relative to untreated controls and increased the ratio of p-ERK/ERK and p-AKT/AKT [124]. This effect was abrogated by pharmacological inhibition of NKA, suggesting that GPNMB ECF both binds to and modulates the activity of NKA and the downstream signaling cascades.

### Progranulin and GPNMB in neurodegeneration

**Fig. 2:**
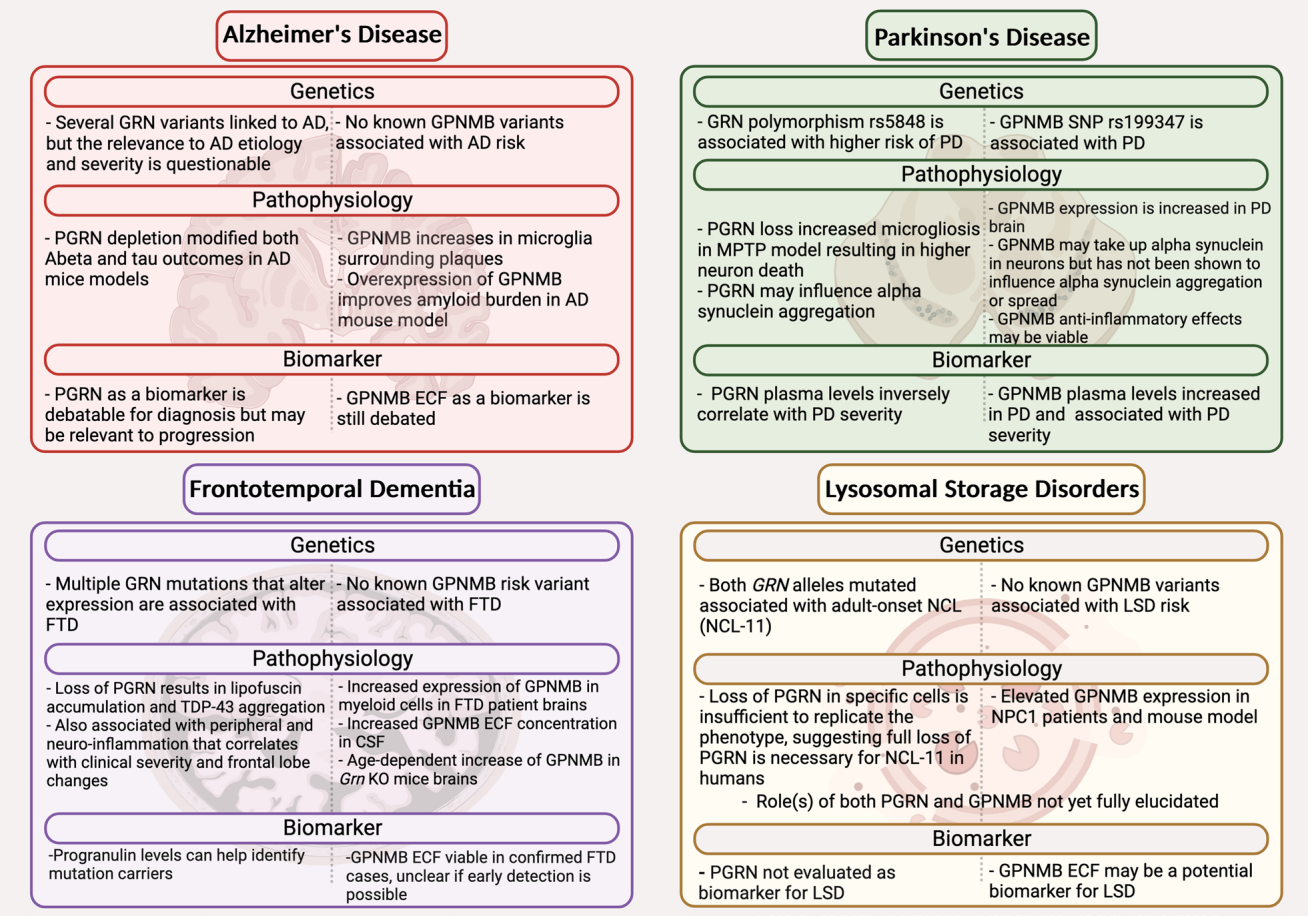
Summary of progranulin and GPNMB in neurodegenerative disease. Figure created with BioRender.com

PGRN has been described as the connecting genetic link between multiple neurodegenerative diseases potentially through regulating inflammation and immunity [6]. Here, a meta-analysis of a published data set from brain gene expression was performed to investigate the extent to which PGRN contributes to AD, PD, and amyotrophic lateral sclerosis (ALS) risk. Interestingly, a significant functional association was identified between increased genetic risk in the *GRN* locus region and decreased PGRN expression in PD, AD, and ALS. The authors concluded that PGRN is involved in the body’s immunological response and could potentially contribute to the etiology of several neurodegenerative diseases [6]. As noted above, while there is no genetic association between *GRN* and ALS, PGRN immunopositivity is increased in microglia in ALS spinal cord [95, 96] and in microglia in spinal cord of mouse models of ALS [97]. The following sections will explore the known effects and interconnections between PGRN and GPNMB in ND with a focus on neuroinflammation and central–peripheral neuroimmune cross-talk (Fig. 2).

### *Frontotemporal dementia (FTD)*

It is known that PGRN is required for healthy lysosomal function throughout the lifespan, because individuals who do not produce enough PGRN of their own are prone to disease. A single defunct *GRN* allele increases the risk of developing FTD, but two mutant alleles result in Neuronal Ceroid Lipofuscinosis (NCL-11), a lysosomal storage disorder (LSD) [125]. Symptoms of FTD can vary widely, even within a single family, but generally include progressive changes in behavior and personality and difficulties with language as well as some motor abnormalities [126]. Accordingly, clinical subtypes categorize FTD patients into behavioral variants (bv) or primary progressive aphasia (PPA), which contain two variants: non-fluent variant (nfvPPA) or semantic variant (svPPA) [126].

Age of onset also varies, but the *R493X* mutation has approximately a 60% penetrance by the age of 60, which increases to 90% by the age of 70 [127]. Histopathological investigation of FTD–*GRN* brains reveals an intracellular and intra-lysosomal buildup of lipofuscin, a pigment that is the result of failed lysosomal metabolism, and an accumulation of TDP-43, a protein involved in RNA management and gene expression [128, 129]. The mechanisms behind TDP-43 aggregation are unclear, but the significant burden of lipofuscin is consistent with other diseases with endo-lysosomal dysfunction.

Another feature of FTD is peripheral inflammation. Given the association of PGRN with inflammation, it is unsurprising that FTD–*GRN* patients exhibit a systemic inflammatory phenotype. One study completed in 2017 demonstrated that PGRN mutation carriers had increased levels of sCD163 and CCL18 in their blood, suggesting increased myeloid cell activation [98]. Furthermore, the amount of LBP correlated with white matter changes in the frontal lobe via MRI [98].

Changes in GPNMB have also been associated with *GRN*–FTD. Bulk RNA sequencing in the cortices of *Grn* KO mice showed an increase in *Gpnmb* mRNA relative to WT control [18]. This was later corroborated with proteomic data also from the cortex of *Grn* KO mice and analysis of human FTD–*GRN* patient brains [17]. These demonstrate an increase in both the RNA and protein of GPNMB in response to insufficient amounts of PGRN. The precise mechanism(s) driving this increase is unclear, but an increase in GPNMB expression has been noted in other varieties of lysosomal disorder, such as Gaucher’s disease (GD) and Niemann–Pick type C (NPC) [55, 60, 130]. Importantly, both pharmacological and gene transfer treatment for GCase deficiency can reverse the increase in GPNMB [55]. This finding suggests that the increase in GPNMB is specific to a compensatory pathway for endo-lysosomal dysfunction and not a result of secondary storage in the lysosome.

### *Lysosomal storage disorders (LSDs)*

Lysosomal storage disorders (LSDs) are a heterogeneous group of heritable (inborn) metabolism defects that affect the function of lysosomes, comprising 70 monogenic disorders of lysosomal catabolism [131]. These disorders are caused by mutations in genes encoding lysosomal proteins, including membrane proteins, transporters, proteases, and lysosomal glycosidases, most of which are inherited as autosomal recessive traits. Mutations in these genes result in lysosomal malfunction and the gradual accumulation of substrates inside the lysosome, leading to cell dysfunction and cell death; these monogenic disorders can be subclassified according to the biochemical type of stored material, such as the sphingolipidoses, mucopolysaccharidoses, and glycoproteinoses. Of interest to this review are the neuronal ceroid lipofuscinoses (NCLs), which are neurodegenerative diseases characterized by the storage of abnormal lipopigment and lipofuscin in lysosomes. NCLs are fatal disorders that are clinically and genetically heterogeneous, with 14 genes implicated to date, (CLN1 through 14), including *GRN *[132].

As previously discussed, the majority of FTD–TDP-causing mutations identified are located in the *GRN* gene [133, 134], although more recently, it has been shown that other NCL-related genes are implicated in FTD cases, and the degree to which *GRN* mutations are identified in FTD-patients is region-specific [135]. Interestingly, it was subsequently reported that a complete loss of *GRN* results in adult-onset NCL [138]. It was reported that two siblings were shown to carry a homozygous deletion of four base pairs in the *GRN* gene (c.813_816del), which lead to a frameshift and premature termination of translation, and previously shown to cause FTD–TDP when inherited as a heterozygous mutation [137–139]. The co-occurrence of FTD and NCL within a family due to *GRN* mutations has been reported and provides the unique opportunity to investigate the genotype–phenotype correlations regarding *GRN* dosage effect; the homozygous relative displayed dysarthria, cerebellar ataxia, retinal dystrophy, and severe global cerebellar atrophy [140]. In contrast, heterozygous relatives presented behavioral variant FTD (bvFTD) and some extrapyramidal features compatible with corticobasal syndrome.

The discovery of the pleiotropic effect of homozygous and heterozygous *GRN* mutations was remarkable, given that the two clinically distinct neurological disorders have very different pathologies. Furthermore, given that the predominant clinical and pathological features of FTD and NCL are distinct, it is controversial whether the disease mechanisms associated with complete and partial PGRN loss are similar or distinct. Given the link between *GRN* mutations and NCL, it has been suggested that impaired lysosomal function may represent an important pathway leading to *GRN*-linked neurodegeneration. Interestingly, iPSC-derived cortical neurons from an FTD patient with a heterozygous *GRN* mutation exhibit neuronal phenotypes similar to characteristic hallmarks of NCL patient pathology, including lipofuscin accumulation and the appearance of fingerprint-like profiles and granular osmiophilic deposits [76]. Similarly, iPSC-derived cortical neurons derived from an NCL patient with homozygous *GRN* mutations exhibit TDP-43 mislocalization, enlarged autofluorescent lysosomes, and electron-dense vesicles containing storage material with granular, curvilinear, and fingerprints profiles [141]. *GRN*-mutant neurons from an FTD patient also showed decreased activity of the lysosomal enzyme cathepsin D via a loss of the PGRN cleavage product, cathepsin E. Interestingly, homozygous *GRN* and *CTSD*, which encodes for cathepsin D, mutations lead to a similar form of NCL [142–144], suggesting this functional relationship may provide a possible mechanism for the overlapping NCL-like pathology observed in *GRN*-neurodegeneration. Similarly, it has been shown that PGRN haploinsufficiency in humans leads to preclinical retinal lipofuscinosis and increased lipofuscinosis and intracellular NCL-like storage material also in the cortex, *postmortem* [125], all of which are NCL-like features and were seen, in some instances, prior to the onset of dementia.

It is clear from such human data that there are perhaps similar mechanisms between FTD and NCL despite the clinical distinctions between the two. However, studies from mice have thus far not been consistent with human data. Mice homozygous for a targeted deletion of the mouse *Grn* gene develop a robust neuropathological phenotype with age, with lipofuscin deposition and accumulation of NCL-like storage material as well as microgliosis and astrogliosis in multiple brain regions [145–149], as well as hyperinflammatory responses in both microglia and peripheral macrophages [25, 99, 150]. However, heterozygous *Grn*-targeted mice do not develop such phenotypes [145, 147], with phenotypes limited to decreased sociability and altered social dominance [151, 152]. It has been suggested that the lack of phenotypes in these mice may be due to these mice containing disrupted *Grn* alleles; therefore, they have limited utility in phenocopying progranulin-deficient FTD and NCL caused by nonsense mutations [153]. Recently, the *Grn*^*R493X*^ mouse model was generated to more accurately model FTD–GRN by introducing one of the most common human nonsense mutations leading to FTD (*R493X*) at the analogous mouse *Grn* codon (*R504X*) [153], as previously discussed. Homozygous *Grn*^*R493X*^ mice phenotypically replicate several neuropathological hallmarks previously demonstrated in *Grn* null mice and have been shown to have lysosomal dysfunction, neuroinflammation, and thalamic neurodegeneration [154]. Notably, evidence of lysosomal dysfunction has been demonstrated in *Grn* null mice as young as 2 months of age [155], while lysosomal dysfunction has only been reported in aged 18-month-old *Grn*^*R493X*^ mice [155]. Future efforts should seek to understand whether the presence of a semi-functional, truncated *Grn*-*R493X* might delay the onset of this early lysosomal phenotype and how these phenotypes from murine models inform researchers of the shared and disparate phenotypes and mechanisms between FTD and NCL.

It has previously been demonstrated that the conditional loss of PGRN in neurons is not sufficient to cause NCL-like neuropathology in mice [156], leading researchers to hypothesize that *GRN* mutations in NCL may exert their disease effect in a cell-autonomous manner and that microglia may be the culprits underlying disease development and progression. However, selective reduction of microglial PGRN in neuronal PGRN-deficient mice failed to induce lipofuscinosis or gliosis observed in *GRN* null mice [157]. These data lead the authors to conclude that PGRN from extracellular sources may prevent pathology. However, PGRN expression is not confined to the brain, and it is clear that many LSDs have a peripheral component. Indeed, evidence suggests that the accepted name of NCLs as ‘neuronal’ may be a misnomer, since it is emerging that the effects of the disease are certainly not confined to neurons or even within the brain. Indeed, NCL proteins, including PGRN, are widely expressed in various tissues and cell types [100, 158–161], and therefore, it is unsurprising that other organ systems in the body are affected by deficiency in these proteins [162]. Indeed, as previously discussed, PGRN loss has recently been described to result in sex-dependent dysregulation of both the peripheral and the central immune system in mice [100], raising the interesting possibility that peripheral inflammation may also play a role in neurodegeneration due to *Grn* haploinsufficiency in human FTD–GRN mutation carriers.

Although the function of GPNMB in modifying NCL pathology has not been investigated in a clinical or pre-clinical settings, it has been shown that GPNMB is elevated in Niemann–Pick disease type C1 (NPC1) mice and patients, a rare and fatal neurodegenerative–LSD that arises from lysosomal accumulation of unesterified cholesterol and glycosphingolipids [130] and may be a potential biomarker for therapeutic trials [163]. In addition, GPNMB expression levels have been shown to be modulated in both central and peripheral myeloid cells in *Grn*-deficient mice, suggesting that PGRN and GPNMB may jointly regulate the peripheral and central immune system, and future research would benefit from further exploring the mechanisms behind this relationship and how it may be utilized as a potential biomarker for *GRN*-associated NDs, including NCL.

### *Alzheimer’s disease (AD)*

AD is a ND characterized by the presence of amyloid beta (Abeta) plaques and tau neurofibrillary tangles, which clinically results in severe cognitive impairment [164]. The link between PGRN and AD are supported by genetics, where several *GRN* mutations in human studies have been associated with increased risk for AD, including a null mutation IVSO + 5G > C, the *GRN* nonsense mutation p.Arg535X [7], two missense mutations, p.Cys139Arg and p.Pro451Leu [8] and the exon 1 *GRN* mutations, p.Gly35Arg and pASP33Asp [9]. Among all, one of the most well-studied *GRN* variants is the rs5848 variant, which has been described in different AD populations [10–12] and has been associated with increased risk for AD by three meta-analyses [14, 165, 166]. However, some discrepancies have been suggested regarding its implications in PGRN expression, where the rs5848 variant translated in the decrease of *GRN* mRNA levels in the parietal lobe and PBMCs of AD patients [10] but also has been described to no altered *GRN* expression in serum levels between controls and AD patients [167].

Importantly, the role of PGRN as a biomarker for AD has been proposed, but it is also controversial. Specifically, *GRN* levels appear to be upregulated at the transcriptomic level in the blood of AD patients in comparison with controls. However, patients that carried the rs5848 variant displayed a decreased *GRN* expression [168, 169]. In contrast, the extent of PGRN protein expression in the blood showed no relationship with disease severity [169]. In addition to measurements in the blood, PGRN levels have been examined in the CSF of AD samples, with discordant outcomes reported. In one study, CSF PGRN levels were highest in AD samples compared with controls and MCI samples and were correlated with cortical thickness [170]; however, in another study, PGRN CSF levels did not differ between AD and non-AD patients and did not correlate with cortical thickness [171]. Importantly, the role of PGRN as a biomarker for AD has been considered due to its relationship to neuroinflammation [172], where CSF PGRN levels have been described to increase and to be associated with an increase in inflammatory markers in tau-neurodegenerative (TN) + patients [173]. Finally, a recent paper suggested that CSF levels of PGRN may represent a marker of AD disease progression rather than a diagnostic marker for AD [174].

A regional characterization of PGRN and GRNs expression in AD, FTD–TDP human brains, and unaffected controls with and without *GRN* mutations has been examined [175]. Here, neuronal PGRN immunopositivity was decreased in AD samples with and without *GRN* mutations, while PGRN was increased in microglia. Furthermore, differences in GRN immunopositivity were found; GRN C was found in microglia-positive cells forming patches in the cortex of control and AD brains, whereas GRN B showed the strongest signal in hippocampal pyramidal neurons [175]. In other studies, PGRN expression has been studied in the context of amyloid plaques. In the middle temporal gyrus, the size and number of PGRN-associated plaques increased in AD samples, and PGRN expression colocalized with microglia (IBA1 + cells) and vasculature (CD31 + cells) but not with astrocytes (GFAP + cells) [176]. In addition, a correlation between increased protein PGRN and Abeta and phosphorylated tau (p-tau) expression was observed [176]; however, there was no colocalization between PGRN and p-tau at cellular levels by immunohistochemistry. This relationship between PGRN and neurofibrillary tangles was further explored in a separate study, where PGRN, and also prosaposin, are decreased in neurons that develop neurofibrillary tangles compared with neurons without them [177].

Considering the findings in human studies, mouse AD models have been used to further understand the consequences of PGRN loss on AD-like phenotypic aspects such as Abeta plaque load, tau phosphorylation, inflammation, or synaptic loss and the protective effects of PGRN replacement in such models. The link between PGRN and AD through Abeta pathology has been explored in mouse models such as Tg2576, APP/PS1, or 5xFAD crossed with mPGRN-deficient mice. In Tg2576 mice, *Grn* has been shown to be upregulated in the hippocampus, and mPGRN immunoreactivity was observed around dense core plaques, which increased in aged mice. In these mice and at the cellular level, *Grn* overexpression was described in microglia, neurons, and neurites around dense plaques [178]. In APP transgenic mice, mPGRN levels decreased in the cortex of 3- and 7-month-old APP^high^ mice and 11–12-month-old APP^low^ mice but increased in 13-month-old 5xFAD mice [179]. In addition, the role of the immune system and, more specifically, of microglia was suggested in the context of APP and PGRN deficiency. For example, *Grn* KO mice crossed with APP^low mice^ displayed differences in microglia burden, with higher CD68 expression in the hippocampus of APP^low^ *Grn* KO relative to APP^low^ WT controls. In addition, the expression of pro-inflammatory cytokines (such as *Tnf* and *Il1b*) were increased, and expression of anti-inflammatory cytokines (such as *Il-4 or Cox2*) was decreased. Finally, the selective depletion of mPGRN in microglia in APP^high^ mice resulted in a 50% increase in hippocampal plaque load, hypothesized to be caused by an impairment of microglia phagocytosis resulting from mPGRN deficiency [179]. A similar finding on microglia was reported in the *Grn* KO x 5xFAD mice model [180]. In this study, mPGRN was shown to be expressed in microglia near Abeta plaques, and mPGRN depletion decreased Thioflavin S-reactive and Abeta plaques in the cortex of 4–5-month-old male mice. Finally, RNA-seq analyses of the hippocampus of 5xFAD mice with or without mPGRN revealed that lysosomal and inflammatory genes such as *Cd68* or *Gpnmb* were involved in this process and, specifically, *Gpnmb* was shown to be upregulated in microglia around Abeta plaques in 5xFAD mice lacking mPGRN [180]. Together, these data indicate that PGRN has a potential role in Abeta deposition and clearance through the immune system, mainly microglial cells; hence, several studies have aimed to increase PGRN levels as a way to improve AD-like pathology in mice [179, 181, 182]. To examine the protective effects of PGRN in pre-clinical models of AD, *Grn* viral delivery has been used in the 5xFAD and the Tg2576 mouse models to increase mPGRN levels. In the 5xFAD mouse model, the increase in mPGRN levels decreased amyloid-beta plaque load, protected against Abeta toxicity, and a decrease in plaque load in the hippocampus and on neurons in the hippocampus was observed [179]. Similarly, Tg2573 mice that received a viral vector delivery of mPGRN (ND-602) displayed reduced amyloid plaque burden in the hippocampus and the entorhinal cortex, a reduction of the inflammatory proteins fluorescein-conjugated isolectin B_4_ (IB4), IBA1 and GFAP in the hippocampus, and a reduction in synaptophysin in the dentate gyrus and hippocampus. In addition, ND-602 gene therapy in Tg2573 increased neprilysin, an Abeta-degrading enzyme [182]. In addition to viral vector delivery, mPGRN levels have been increased in AD models using an intrahippocampal injection of mPGRN in the 5xFAD mouse model [181]. In this study, the injection of mPGRN to 5xFAD mice decreased amyloid deposition in the hippocampus of 5XFAD mice and decreased BACE1, which is responsible for the APP processing and generation of Abeta peptides. Finally, differences in neuroinflammation were observed after mPGRN intracerebral injection in 5xFAD mice, with IBA1 + cells colocalizing with Abeta plaques, and CD68/IBA1 area were both increased with PGRN injection [181]. Taken together, mPGRN deficiency models and strategies to replace or overexpress mPGRN in mouse models support its role in mitigating neuroinflammation and progression of amyloid-associated pathology.

In addition to Abeta, the relationship between PGRN and tau has also been studied in pre-clinical models [183]. P301L tau mice crossed with *Grn* KO mice revealed that tau phosphorylation was increased in the Tris-saline soluble fraction of 13-month-old, and the sarkosyl-insoluble fraction of 19-month-old P301L tau mice crossed with mPGRN-deficient mice [183]. Interestingly, an opposite effect on Abeta and tau was reported in APP/PS1 mice deficient in mPGRN, which displayed reduced diffuse amyloid-beta plaque deposition and increased neuronal injury [184]. Additional studies to clarify the role of mPGRN in the setting of amyloid pathology with and without accompanying tau pathology will be needed.

Presently, no known GPNMB mutations are associated with an altered risk for developing AD, but a histopathological study of AD brains showed a significant increase in GPNMB-positive staining in the frontal cortex relative to control [185]. The GPNMB-positive staining primarily colocalized with microglia cell markers, and the GPNMB-positive microglia were clustered around Abeta deposits and phosphorylated tau pathology, suggesting that GPNMB expression is associated with microglial activation [185]. This result is consistent with previous work showing increased GPNMB expression in the brains of sporadic AD patients, which localized around both amyloid plaques and blood vessels [186].

The same study also investigated the role of GPNMB on amyloid-associated pathology in the 5xFAD mouse model. One interesting observation was an age-dependent increase in cerebral *Gpnmb* mRNA beginning at 7 months of age in the 5XFAD mice relative to WT controls. A similar effect in cerebral *Gpnmb* mRNA was observed in the APP/PS1KI model relative to PSKI animals but was absent in the APP23 mouse model [186]. Immunofluorescent staining of 12-month-old 5xFAD mice brains showed GPNMB colocalizing with IBA1 but not with GFAP or NeuN, confirming the increase in GPNMB occurred predominantly in myeloid cells [186], which may consist of both microglia and infiltrating peripheral monocytes. It was also demonstrated that GPNMB-positive microglia in 12-month-old 5xFAD mice localize around amyloid plaques, consistent with the findings in human AD patient brains, yet age-matched APP23 mice showed no positive GPNMB staining [186]. These findings suggest that differences in GPNMB expression may be model dependent and potentially associated with different levels of inflammation at different states of pathology.

Recent work has been aimed at investigating the effects and significance of increased GPNMB expression in both AD patient brains and 5xFAD mouse brains. Lentivirus vector overexpression of GPNMB in the brains of APP/PS1 mice rescued Morris Water Maze performance in APP/PS1 mice [187], suggesting that the observed increases in GPNMB in some models of AD-like neurodegeneration may be adaptive or protective and not maladaptive or detrimental. In addition, immunohistochemical analysis of the GPNMB overexpression mouse group revealed decreased amyloid deposition relative to the control mouse group that received the empty vector. This decrease in amyloid deposition was correlated with alterations in LC3-II, p62, and mTOR phosphorylation levels in hippocampal lysate from GPNMB-overexpressing mice [187]. Further mechanistic work in BV2 microglia cells confirmed the changes in LC3-II, p62, and mTOR phosphorylation and further demonstrated that GPNMB overexpression resulted in increased phagocytosis of Abeta relative to empty vector controls, and this effect was abrogated with the addition of an autophagy inhibitor, 3-MA [187]. These findings suggest that GPNMB modulates increases in autophagy, which may be the mechanism responsible for the associated decrease in Abeta deposition.

Given that the extracellular domain of GPNMB can be shed as an extracellular soluble fragment (ECF), it is a potential biomarker candidate for neurological disease, including AD, but different groups disagree as to whether GPNMB is a reliable biomarker of disease progression. A paper published in 2020 compared multiple sets of AD CSF proteomes with their own two-dimensional liquid chromatography fractionation and high-resolution tandem mass spectrometry (abbreviated as TMT–LC/LC–MS/MS) [188]. Here, GPNMB ECF was detected in their own mass spec data, but GPNMB ECF only overlapped with one of the three CSF proteomes that were used to compare expression [188]. Follow-up analysis with an ELISA showed an increase in GPNMB ECF in the CSF of AD patients relative to non-AD controls, and the concentration of GPNMB ECF correlated with the spectrometry data from the same CSF sample [188]. Given their detection and confirmation of GPNMB ECF in the CSF of AD patients, the authors contend that GPNMB is a viable biomarker for AD but caution that less sensitive approaches to mass spectrometry may miss a majority of the proteins detected in the AD CSF proteome. In contrast to that work, a paper published in 2021 found no difference in GPNMB ECF concentration in the CSF of AD patients versus healthy non-demented controls [65]. It should be noted that these papers used different methods to detect GPNMB ECF in addition to having studied different cohorts of AD patients with dissimilar disease stage. At present, there is no definitive conclusion about the reliability of GPNMB ECF as a viable and specific biomarker for AD and future studies will be needed to better understand its role in AD and potential as a biomarker.

### *Parkinson’s disease (PD)*

PD is a neurodegenerative motor disorder characterized by the loss of dopaminergic neurons in the nigrostriatal pathway and the presence of alpha-synuclein (α-syn) inclusions [189]. Although the majority of research regarding the role of PGRN in neurodegeneration has been focused on FTD, LSDs, and AD, there is mounting evidence linking the importance of PGRN function with development of PD. In 2007, Leverenz et al. used a combination of neuropathological and molecular genetic approaches to phenotype two families with the *GRN* mutation c.709-2A > G which reduces *GRN* mRNA expression [13]. In this study, patients that carried this specific PGRN mutation exhibited language impairment, behavior disturbances, and parkinsonism as clinical manifestations, probably explained by the loss of neurons in the neocortex, striatum, hippocampus, and substantia nigra, respectively. In addition, gliosis was observed in the striatum and substantia nigra of the affected patients relative to controls implicating PGRN in modulation of immune responses associated with PD pathophysiology. Interestingly, six cases exhibited ubiquitin/TDP43 immunopositive inclusions, all but two had tau pathology, and two of them exhibited α-syn inclusions, suggesting that this specific *GRN* mutation does not only contribute to the development of FTD but also to parkinsonism and α-syn pathology [13].

More recently, a meta-analysis was performed in 16 case study to investigate the link between the *GRN* polymorphism at rs5848 with neurodegenerative diseases, such as FTD, AD, PD, and ALS [14], and the authors conclude that the polymorphism at rs5848 is associated with a higher risk of AD and PD. Supporting this, reduced PGRN plasma levels have been associated with PD severity [34], where PGRN measured by ELISA negatively correlates with the PD severity and disease duration measured by the Unified Parkinson’s Disease Rating Scale scores (UPDSR-III) [34]. Collectively, therefore, it seems PGRN expression and function may be implicated in modulation of PD onset and/or progression, although the precise mechanism linking this to disease is currently unknown, but we posit is associated with neuroinflammation and lysosomal dysfunction that contributes to α-syn pathology.

The role of PGRN in modulation of PD onset and progression have been studied in human patients and mouse models of PD-like pathology. In this context, Martens et al. investigated whether the putative anti-inflammatory properties of PGRN [151] contributed to neurodegeneration in a PGRN-deficient setting by exacerbating neuroinflammation. The authors used mPGRN-sufficient mice (*Grn* + / +) and mPGRN-deficient mice (*Grn* KO) treated with MPTP, a known neurotoxin that affects the dopaminergic neurons in the substantia nigra *pars compacta* (SNpc). The authors observed that mPGRN-deficient mice exhibited an increase in MPTP-induced neuronal loss in the SNpc and that this effect was mitigated in MPTP-treated mPGRN-deficient mice after mPGRN replacement via lentivirus [151]. In addition to this and using conditional mutants lacking mPGRN in microglia, the authors confirmed that the loss of dopaminergic neurons in the SNpc is driven by microgliosis and not because of the selective vulnerability of neurons due to an increase of activated microglia in mPGRN-deficient mice treated with MPTP [151]. Together, these important findings suggest that, in response to oxidative stress, mPGRN-deficient microglia and/or other myeloid cells adopt a hyperactive pro-inflammatory state, contributing to neuronal loss. Hence, mPGRN gene delivery seemed like a reasonable neuroprotective strategy in the MPTP model. Using a unilateral intranigral infusion of the lentiviral vector ND-602 encoding full-length mPGRN to efficiently transfect nigral neurons in MPTP-treated B6 mice, the neuroprotective effects of mPGRN gene therapy were demonstrated as measured by reduced locomotor deficits, including decreased akinesia/bradykinesia, and increased locomotion velocity and coordination [190]. ND-602 treatment also ameliorated neuronal death in the SNpc and striatum and exerted anti-inflammatory properties as measured by attenuated expression of IB4-positive microglial cells in comparison with mice treated with MPTP but not ND-602 [190]. Finally, a potential link between PGRN expression in microglia and α-syn burden has also been observed. In this context, mPGRN protein was reported to be decreased in mouse microglia exposed to α-syn ex vivo and in human microglia surrounding α-syn deposits [191], suggesting that ingestion of α-syn may negatively regulate mPGRN protein expression in microglia which could result in dysregulated phagocytosis and inflammatory responses. Taken together, all these studies implicate PGRN in development and progression of PD, most likely through modulation of inflammatory responses; yet the specific mechanisms by which this occurs have yet to be explored and will be needed to inform on the therapeutic potential of PGRN replacement in sporadic PD and other parkinsonisms.

Just like for PGRN, the link between GPNMB and PD has been explored in human samples and in mouse models of PD-like pathology. For instance, a single nucleotide polymorphism (SNP) in GPNMB at rs199347 is associated with PD risk [15, 18], and GPNMB expression has been reported to be increased in the SNpc of PD patient brains post-mortem [124, 192, 193]. In a paper published in 2018 by Moloney et al., the authors investigate two pathogenic mechanisms by which GPNMB contributes to PD; changes associated with lysosomal dysfunctions and regulation of glycolipids (in a CBE (conduritol-ß-epoxide) mouse model) or α-syn (in the Thy1-ASYN mice) [192]. In the CBE mouse model, GPNMB was increased in brain regions, such as the motor cortex, hippocampus, and SNpc upon CBE treatment. By contrast, in the brain of α-syn-overexpressing mice (Thy1–ASYN mice), no differences in GPNMB expression were found in any of the studied brain regions, indicating that GPNMB may contribute to PD pathology indirectly through lipidopathy changes [192].

Another recent study independently investigated the link between the rs199347 SNP and PD using a combination of computational, cell biological studies, and clinical samples [194]. Here, the rs199347 variant was associated with higher GPNMB expression in the brain and blood. In addition, using human pluripotent stem cells-derived cortical neurons expressing different levels of GPNMB, a key role of GPNMB in synaptic defects was suggested, where GPNMB was reported to be required for α-syn pre-formed fibril (PFF) uptake by neurons; importantly, its potential role in immune cells was not explored [194]. Interestingly, interrogation of the role of GPNMB in α-syn biology using mouse models has yielded controversial results. In this context, the work published by Brendza et al. showed that GPNMB loss did not modify changes in a remyelination mouse model or in two different mouse models of PD-like α-syn pathology (synthetic human α-syn PFFs and AAV1/2-CMV/CBA virus vector driving human mutant A53T α-syn) [195]. With regards to the α-syn models, human α-syn PFF injections into the striatum were employed to evaluate the differences in α-syn spreading and aggregation, motor dysfunction, and gene expression between WT and GPNMB-deficient mice. While α-syn PFF aggregation caused α-syn spreading in the midbrain, striatum, and brainstem, neurodegeneration in the midbrain and brainstem, and both astrogliosis and microgliosis, no difference was found in PFF-treated mice that lacked GPNMB compared to PFF-treated WT mice. In addition to these neuropathological measures, behavior studies such as wire hang and open field showed no differences in mice lacking GPNMB. Finally, RNA-sequencing from the striatum, midbrain, and brainstem revealed that *Gpnmb* was the only differentially expressed gene (DEG) that was identified between WT and *Gpnmb* KO PFF injected mice, indicating that at the transcriptional level, there were also no differences between WT and GPNMB-deficient mice treated with PFFs [195]. Similar findings were observed in a second α-syn model, where the over-expression of human mutant A53T by AAV in WT and GPNMB-deficient mice showed no differences in TH loss, and *Gpnmb* was again the only DEG upregulated gene in midbrain and striatum in RNA-seq analyses [195]. Together, these surprising findings indicate that, at least in two mouse models of α-syn pathology, GPNMB is not required to protect against it. An alternative interpretation is that global GPNMB deficiency in mice resulted in genetic compensation that rendered neurons resistant to α-syn-induced pathology. Additional studies on the role of GPNMB using acute knockdown strategies in different cell types will be needed to distinguish between these and other possibilities. Finally, Budge et al. explored the possibility of using *Gpnmb* overexpression as an anti-inflammatory regimen to prevent neuronal loss in the MPTP mouse model [196], where it was shown that transgenic overexpression of *Gpnmb* protects against dopaminergic neuron loss. In the context of neuroinflammation, at the gene expression level, the overexpression of *Gpnmb* in MPTP-treated mice reduced *Aif1* and *Gfap* expression in the striatum and primary microglia treated with recombinant GPNMB and LPS increased the expression of anti-inflammatory genes, such as *Arg1, Mrc1, Nrf2, Ym1*, and *Jmjd3*, which are downregulated in LPS-treated WT microglia [196]. Together, these findings suggest that *Gpnmb* overexpression can exert protective anti-inflammatory effects. Additional studies will be needed to reconcile differences in the protective effects of GPNMB that appear to be model dependent and/or dependent on global versus conditional deletion of GPNMB in relevant cell types.

### Summary of animal models

A great deal of the data discussed in this review was generated using Grn KO mouse lines to interrogate the consequences of progranulin-deficiency. However, there are slight differences in how each knockout line was generated. To succinctly present the differences, we produced a table that contains the commonly utilized *Grn* KO mouse lines, the differences in the manipulation of the *Grn* gene, as well as brief summaries of notable findings that demonstrate endo-lysosome dysfunction and altered inflammation and immune cell phenotypes (Table 1).

**Table 1:** Summary of PGRN KO mouse lines and their endo-lysosomal and immune cell phenotypes

Mouse line (nickname)	Gene manipulation	Endo-lysosomal dysfunction	Inflammation and immune cell function
*Grn* KO (Nishihara)	Targeted deletion of exons 2–13 of *Grn* gene [209]	• Increase in *Lyz2* and *Ctsz* in hippocampus with LPS treatment [197] • Increased Lamp1 + and mature CtsD + in immortalized cell lines and primary microglia [73] • Increased LAMP1, saposin D, CTSD, and TMEM106b in whole brain lysate [199] • Increased autofluorescent material in retinal lysosomes [200] • Age-dependent increase in Lamp1 + and p62 + staining, *Tfeb*, *CtsD*, and *Atp6v0d2* transcript, and autofluorescent signal in cortex, thalamus, and VPM/VPL [201] • Increased *Cd63*, *Cd68*, *Hexb*, and *Ctsd* expression in brain [202]	• LPS-mediated increase in Cd68 + staining, increase in *Mpeg1*, *Il-1b*, and *mPges-1* in hippocampus [197] • Increase in IBA1 + cells around choroidal neovascularization lesion. Increase CD68 + macrophage cell line in laser-irradiated RPE–choroid complex [198] • Age-dependent increase in Iba1 + and Cd68 + cells. Increase in *Tnf*, *Mpeg1*, *Cyba*, *Cybb*, *C4*, and *Lcn2* expression in cortex, thalamus and VPM/VPL [201] • Increased serum Il-6, Il-10, and MCP1 after LPS, Decreased Listeria/Lc3 co-localization in *Grn* KO BMDM [128]
*Grn* KO (Ding)	Targeted deletion of exons 1–4 of *Grn* gene [99]	• Prosaposin trafficking defects in immortalized cell lines and brain [35] • Age-dependent increase in Gpnmb and Galectin-3, primarily on microglia [17] • Increased Galectin-3 +, Plin2 +, and lipofuscin accumulation in response to demyelination in brain [205] • Increased Lamp1 +, Cathepsin D +, and lipofuscin accumulation [206] • Decreased abundance of BMP species, age-dependent increase in glucosylsphingosine and decrease in GCase activity in brain and BMDM [207]	• Increased Tnf, Il-6, and iNos and decreased Il-10 after spinal cord injury [203] • Increased *Tnf, Il-6,* and *Il-1b* after LPS treatment in macrophages [99] • Increase Tnf, Il-6, and *Il-1b* after LPS injection, increased immune cell infiltration into lungs during LPS treatment [204] • Age-dependent increase in plasma Gpnmb ECF [17] • Increased Iba1 +, Gpnmb +, and Trem2 + staining in response to demyelination in brain [205] • Increased Iba1 +, Gfap +, and Cd68 + [206] • Sex-dependent effects in immune cells: increase in Ly6C high monocytes in blood, decrease of MHCII expression in microglia, increase in CD8 + T cells in both blood and brain, altered CD44 expression on T-cell populations [100]
*Grn* R493X (knock-in)	Knock-in of R504X *Grn* gene; mimics the R493X *GRN* mutation, positive for nonsense-mediated decay [153]	• Altered ganglioside metabolism and decreased BMP levels in brain [82] • Increased cathepsin D expression, higher Lc3II/LC3I ratio in brain [154]	• Increased Iba1 +, GFAP +, and C1q + staining in brain [154] • Age-dependent increase *Il-1b*, *Tnf*, and *Mcp1* transcript [208]
Floxed *Grn*	loxP sites flanking the *Grn* coding sequence; complete removal of *Grn* gene when crossed with *Cre* line, allows for cell-specificity [150]	• Altered lipidomic profile with successive loss of *Grn* alleles. Increased expression of *Ctsd*, *Cd68*, and *Lyz2*, and Increased number and size of lysosomes in neurons [83] • Increased β-Gal, β-Hex, HexA, GLA and GCase activity [78] • Age-dependent increase in pro- and mature CstD and Lamp1 [211]	• Increased mRNA of *Tnf*, *Il-1b*, and *Il-6*; decreased *Il-10* [150] • Increased expression of *Trem2* and *Tyrobp* [83] • Increased Iba1 + and Gfap + staining in the hippocampus, cortex, and thalamus [152] • Increased Iba1 + staining in the thalamus [210]

### Therapeutics in development

Currently, no disease-modifying treatments exist for FTD–*GRN* mutation carriers. Prior to onset, *GRN* mutation carriers maintain the same level of circulating PGRN as symptomatic FTD patients [32], but do not experience the behavioral changes and language difficulties associated with FTD. This highlights the role of aging in the development of FTD–*GRN* and suggests that a compensatory mechanism(s) exists that prevents or delays the progression of disease development. Successful identification of these mechanism(s) will enable targeted intervention to extend therapeutic windows or augment disease-modifying treatments for FTD–*GRN* mutation carriers.

The most logical approach to treat diseases rooted in PGRN deficiency would be to supplement the amount of PGRN or GRNs in the patient, and therefore, multiple therapeutics are in development to do exactly this. Denali Therapeutics has developed a protein transport vector progranulin (PTV–PGRN or DNL593) that is a full-length human PGRN protein attached to a transferrin ligand. This allows the human PGRN to bind to the transferrin receptor, which promotes the successful uptake of the PGRN protein into the CNS. This approach was shown to be effective in reversing the glial reactivity, BMP deficiency, and lipofuscin storage in a *Grn* KO mouse model [207]. Importantly, the authors also demonstrated similar rescue in mice dosed every week versus every other week, suggesting that the rescue may be, at least in part, mediated by the more stable lysosomal-resident GRNs thereby persisting over time without requiring more frequent dosing of PGRN. DNL593 is currently under clinical trials in patients carrying *Grn* mutations (NCT05262023) [212]. The potential for GRN-mediated therapeutic replacement was specifically explored using rAAV-mediated overexpression of human GRN2 or GRN4 in *Grn* KO mice. In a recent pre-print, the authors reported full-length PGRN was not required to rescue lipofuscinosis, microgliosis, and lysosomal function in *Grn* KO mice, and these two individual GRNs rescued to similar but not identical extents [213]. These exciting findings raise the possibility that direct therapy with GRNs may be an additional therapeutic approach in PGRN/GRN-deficient states. An alternative PGRN replacement therapeutic consists of gene therapy. Developed independently by Prevail Therapeutics and Passage Bio, PR006 and PBFT02 are adeno-associated virus (AAV)-based therapeutics that leverage a viral vector to selectively infect the CNS with a viral vector encoding PGRN [214]. This allows for PGRN to be generated in the CNS without the need for uptake and transport from the periphery and may be therapeutically efficacious after a single injection into the cisterna magna [214]. This approach has been used in human IPSC models and mouse models of PGRN deficiency and has shown significant rescue of PGRN levels [211, 215]. Finally, both are in clinical trials with outcome measures focused on the successful expression of PGRN, tolerance for the AAV vector, and neurological and neurocognitive improvements from baseline levels (NCT04408625 and NCT04747431) [216, 217]. Finally, ARKD-104, developed by Arkuda Therapeutics, is labeled a “PGRN enhancer” [214]. ARKD-104 is a small molecule drug that increases PGRN and GRN products and is capable of penetrating the brain [214]. However, the precise mechanism is unknown and may not be tolerated in all cases at an effective dose.

AL001, also known as latozinemab, is another approach to altering progranulin levels. Co-developed by Alector and GlaxoSmithKline (GSK), latozinemab is an antibody specific for the sortilin receptor designed to block full-length PGRN uptake [214]. In brief, the interruption of the PGRN–sortilin interaction prevents the internalization of PGRN and its traffic through the endo-lysosomal system, where it is cleaved into GRNs in the lysosome. The net effect of this inhibition is an increase in the amount of extracellular PGRN, while the intracellular PGRN remains fairly stable [20]. It is believed that sufficient amounts of PGRN are able to intracellularly traffic to the lysosome via non-sortilin-binding partners, such as the M6PR or LRP1, preventing lysosomal dysfunction, while the increased extracellular PGRN can be taken up by neurons or other cell types, where expression is relatively low. The PGRN therapeutics and their mechanisms of action have been summarized in the table below (Table 2).

**Table 2:** Summary of PGRN therapeutics and their mechanisms of action

PGRN Therapeutics	Mechanism of action
DNL593	Direct replacement with brain-penetrant progranulin construct
PR006	Recombinant Adeno-associated virus (rAAV)-mediated gene therapy to increase progranulin expression in the CNS
PBFT02	Recombinant Adeno-associated virus (rAAV)-mediated gene therapy to increase progranulin expression in the CNS
ARKD-104	Small molecule “progranulin enhancer”; full mechanism not described
AL001 (Latozinemab)	Blocks sortilin-mediated trafficking of progranulin. Increases extracellular progranulin without compromising intracellular levels

Regardless of the method, multiple concerns with increasing PGRN levels as a therapeutic approach exist. Toxicity from increased PGRN is the primary concern. While necessary for endo-lysosomal health during aging, PGRN in excess could become toxic and contribute to hyperplasia of certain tissues [218–221]. Furthermore, the AAV vectors must also be tolerated by patients’ immune systems. Poor tolerance of AAV would make some candidate therapies not viable for every patient group. Similarly, off-target effects from small molecule drugs and immunotherapy are possible and must be carefully considered before widespread use in the clinic.

GPNMB as a therapeutic target has not been explored in the field of neurodegeneration, but in oncology, GPNMB has been used as a therapeutic for osteosarcoma and squamous cell carcinoma [222–224], but the efficacy of this therapy is questionable [222, 223]. Previous work has shown that GPNMB increases in response to endo-lysosome dysfunction [55, 117], and decreases with the resolution of the endo-lysosomal challenge [55], which is consistent with GPNMB expression as a compensatory mechanism to aid in endo-lysosome dysfunction. This would argue for enhancing GPNMB expression and/or function in vulnerable cell types, perhaps at greater levels or earlier timepoints than biology would eventually reach unaided, rather than decreasing GPNMB. However, care must be taken to avoid unintended immunomodulatory effects given the role of GPNMB in immune cell communication and coordination.

## Conclusions and future directions

The main objective of this review was to present the current biological knowledge on PGRN and GPNMB interactions within the context of neuroinflammation and neurodegenerative disease. A critical biological function of both proteins is believed to be their role in lysosomal health. Therefore, it is reasonable to expect that mutations or exposures which reduce the levels of these proteins may contribute to dysfunction of endo-lysosomal traffic which would have a negative impact on effector function in the immune system. If this prediction is correct, it is important to ascertain the mechanisms by which these two genes and their protein products interact to develop sound therapeutics for the clinic.

The fact that dysfunctions in the lysosome and the immune system are considered the main pathological mechanisms contributing to ND makes PGRN and GPNMB exciting and important candidates to study in the context of ND. Indeed, we have reviewed the implications of both PGRN and GPNMB in NDs, including FTD, LSDs, PD, and AD, where they separately have been described to be biomarkers of disease and/or to directly contribute to crucial neurodegenerative processes, such as protein aggregation, neuroinflammation, and neuronal loss. However, the specific role of PGRN–GPNMB interactions at the lysosome to regulate lysosomal health and inflammatory responses and how these contribute to NDs needs further investigation.

Taking this together opens up the possibility that the modulation of PGRN and/or GPNMB could serve as a powerful and effective therapeutic approach against NDs. In this context, different therapies are in place targeting PGRN, where its increased expression has been suggested to be protective. However, little has been explored considering GPNMB as a potential therapeutic venue linked to PGRN deficits. Based on our current and emerging knowledge, we expect that more in-depth knowledge of PGRN/GPNMB interplay will result in more effective mechanism-based therapeutics, where targeting PGRN and GPNMB will impact both neurons and neuroimmune health overall as preventive mechanisms towards neuronal loss.

## Data Availability

Not applicable.

## Abbreviations

PGRN, Progranulin; mPGRN, mouse: Progranulin
NDs: Neurodegenerative diseases
FTD: Frontotemporal dementia
AD: Alzheimer’s disease
PD: Parkinson’s disease
LSDs: Lysosomal storage disorders
GPNMB: Glycoprotein non-metastatic B
GRNs: Granulins
ER: Endoplasmic reticulum
SLPI: Secretory lymphocyte precursor inhibitor
NMD: Nonsense-mediated decay
SRP: Signal recognition peptide
UTR: Untranslated region
A9D: Aspartic acid at position 9
PTM: Post-translational modifications
uORF: Upstream open reading frame
miR: Micro RNAs
ECD: Extracellular domain
hemITAM: Half immunoreceptor tyrosine-activation motif
ECF: Extracellular fragment
sGPNMB: Soluble GPNMB
APCs: Antigen-presenting cells
CNS: Central nervous system
MITF: Microphthalmia-associated transcription factor
bmMSC: Bone-marrow mesenchymal stem cells
V-ATPase: Vacuolar-type-H + ATPase
GCase: Beta-glucocerebrosidase
HexA: B-hexosaminidase A
BMP: Bis(monoacylglycerol)phosphate
ASM: Acid sphingomyelinase
LPLA2: Lysosomal phospholipase A2
ALIX: Apoptosis linked gene 2 interaction protein X
NPC2: Niemann–Pick disease type C2 protein
Hsp70: Heat shock protein 70
KO: Knock-out
WT: Wild type
NSCs: Neuronal stem cells
LBP: Lipopolysaccharide-binding protein
LPS: Lipopolysaccharide
TNFR1/2: TNF receptors 1 and 2
YB-1: Y-box-binding protein
BMDMs: Bone-marrow-derived macrophages
pNFkB: Phosphorylated nuclear factor kappa-light-chain enhancer of activated B cells
EMT: Epithelial–mesenchymal transition
GCS: Glucosylceramide synthase
NND: Non-neurological disease
DAM: Disease-associated associated microglia
NKA: Na +/K + ATP-ase
bv: Behavioral variants
PPA: Primary progressive aphasia
nfvPPA: Non-fluent variant
svPPA: Semantic variant
MRI: Magnetic resonance imaging
NPC: Niemann–Pick type C
NCLs: Neuronal ceroid lipofuscinoses
NPC1: Niemann–Pick disease type C1
SNpc: Substantia nigra *pars compacta*
PFFs: Pre-formed fibrils
DEGs: Differential expressed genes
AAV: Adeno-associated virus
GSK: GlaxoSmithKline
M6PR: Mannose-6 phosphate receptor
LRP1: Lipoprotein receptor-related protein 1
